# Supplementary material for: The genetic and biochemical determinants of mRNA degradation rates in mammals
Source: Genome Biol. 2022 Nov 23;23:245. doi: 10.1186/s13059-022-02811-x (PMC9684954; doi:10.1186/s13059-022-02811-x)
Supplement: Supplementary file 1 — Additional file 1: Figures S1-S8. Supplementary figures S1 to S8. [file 13059_2022_2811_MOESM1_ESM.pdf]

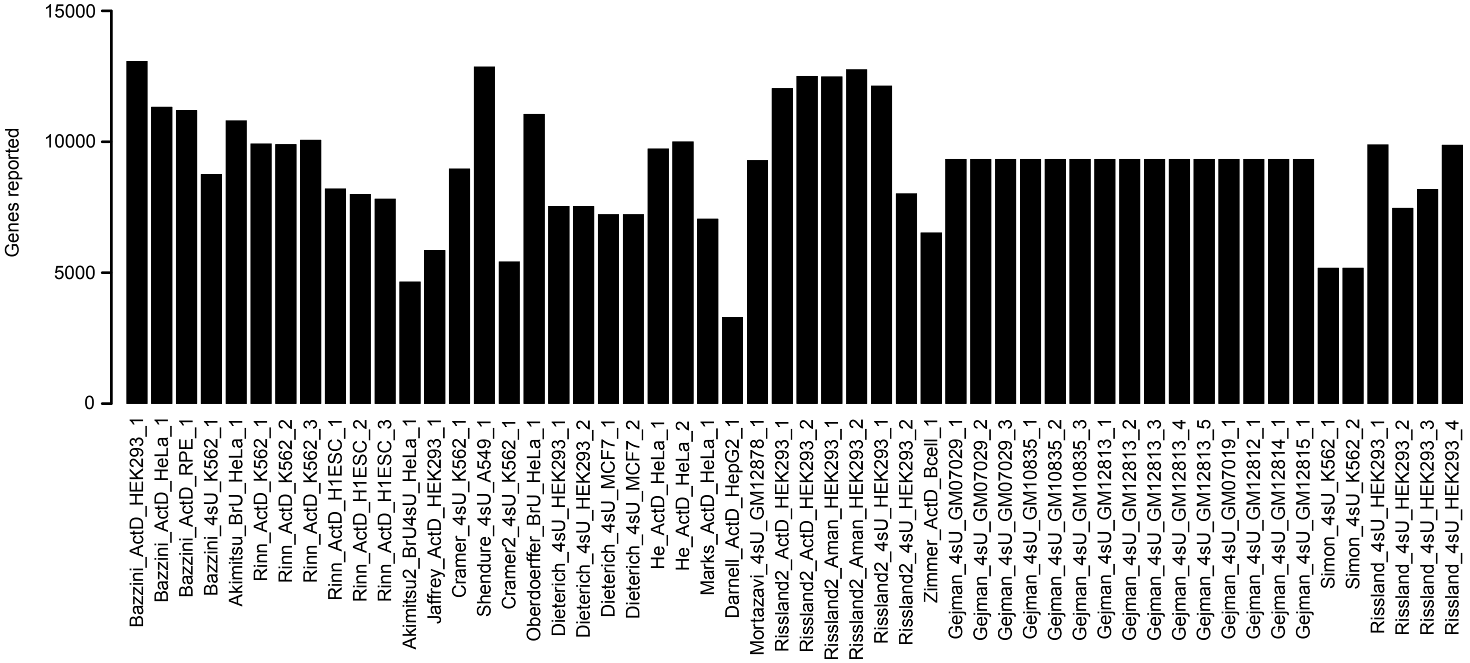

Fig S1. Variability in genes reported in different human half-life datasets.
Barplot of the number of genes whose half-lives were reported in each of 54 human samples. Each sample ID is delimited by underscores and listed according to its study of origin (**Table 1**), measurement method, cell type, and replicate number.

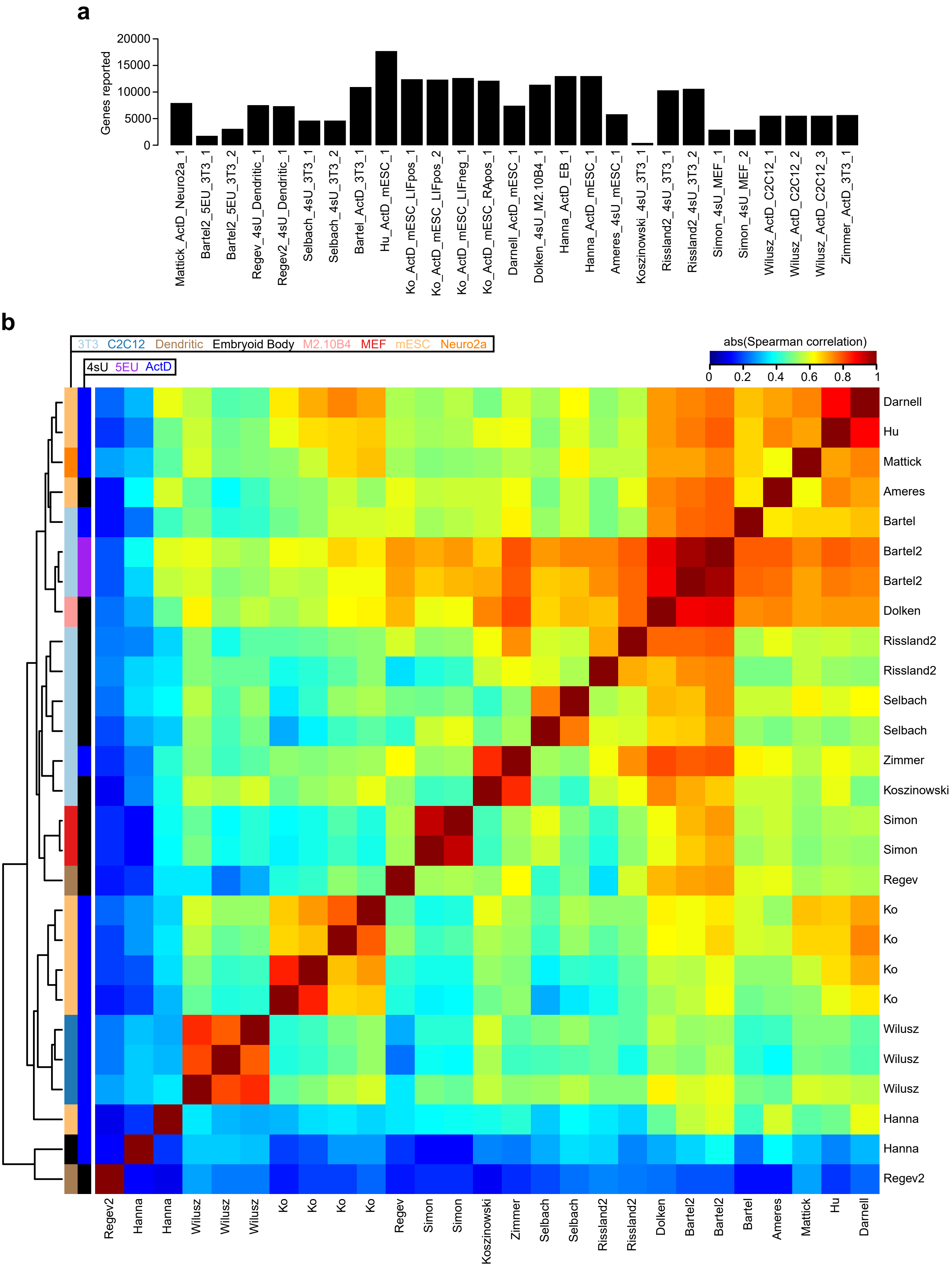
a
Genes reported
20000
15000
10000
5000
0
Mattick_ActD_Neuro2a_1
Bartel2_5EU_3T3_1
Bartel2_5EU_3T3_2
Regev_4sU_Dendritic_1
Regev2_4sU_Dendritic_1
Selbach_4sU_3T3_1
Selbach_4sU_3T3_2
Bartel_ActD_3T3_1
Hu_ActD_mESC_1
Ko_ActD_mESC_LIFpos_1
Ko_ActD_mESC_LIFpos_2
Ko_ActD_mESC_LIFneg_1
Ko_ActD_mESC_RApos_1
Darnell_ActD_mESC_1
Dolken_4sU_M2.10B4_1
Hanna_ActD_EB_1
Hanna_ActD_mESC_1
Ameres_4sU_mESC_1
Koszinowski_4sU_3T3_1
Rissland2_4sU_3T3_1
Rissland2_4sU_3T3_2
Simon_4sU_MEF_1
Simon_4sU_MEF_2
Wilusz_ActD_C2C12_1
Wilusz_ActD_C2C12_2
Wilusz_ActD_C2C12_3
Zimmer_ActD_3T3_1
b
3T3 C2C12 Dendritic Embryoid Body M2.10B4 MEF mESC Neuro2a
4sU 5EU ActD
abs(Spearman correlation)
0 0.2 0.4 0.6 0.8 1
Darnell
Hu
Mattick
Ameres
Bartel
Bartel2
Bartel2
Dolken
Rissland2
Rissland2
Selbach
Selbach
Zimmer
Koszinowski
Simon
Simon
Regev
Ko
Ko
Ko
Ko
Wilusz
Wilusz
Wilusz
Hanna
Hanna
Regev2
Regev2
Hanna
Hanna
Wilusz
Wilusz
Wilusz
Ko
Ko
Ko
Ko
Regev
Simon
Simon
Koszinowski
Zimmer
Selbach
Selbach
Rissland2
Rissland2
Dolken
Bartel2
Bartel2
Bartel
Ameres
Mattick
Hu
Darnell

Fig S2. Comparison of half-lives in a compendium of mouse datasets.

a) Barplot of the number of genes whose half-lives were reported in each of 27 mouse samples. Each sample ID is delimited by underscores and listed according to its study of origin (**Table 1**), measurement method, cell type, and replicate number. **b)** Heatmap of the absolute value of the Spearman correlations measured between half-lives derived from each pair of 27 mouse samples. Samples are clustered using hierarchical clustering according to the indicated dendrogram. Rows are labeled by the study of origin (**Table 1**) and colored by the cell type of origin and measurement approach.

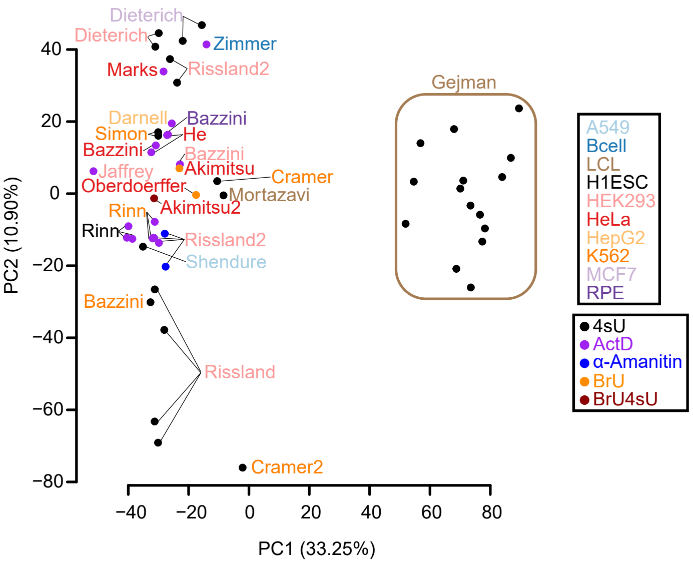

Fig S3. Relationship among human samples.
This figure is similar to **Fig. 2a**, except that it shows all human samples evaluated in this study.

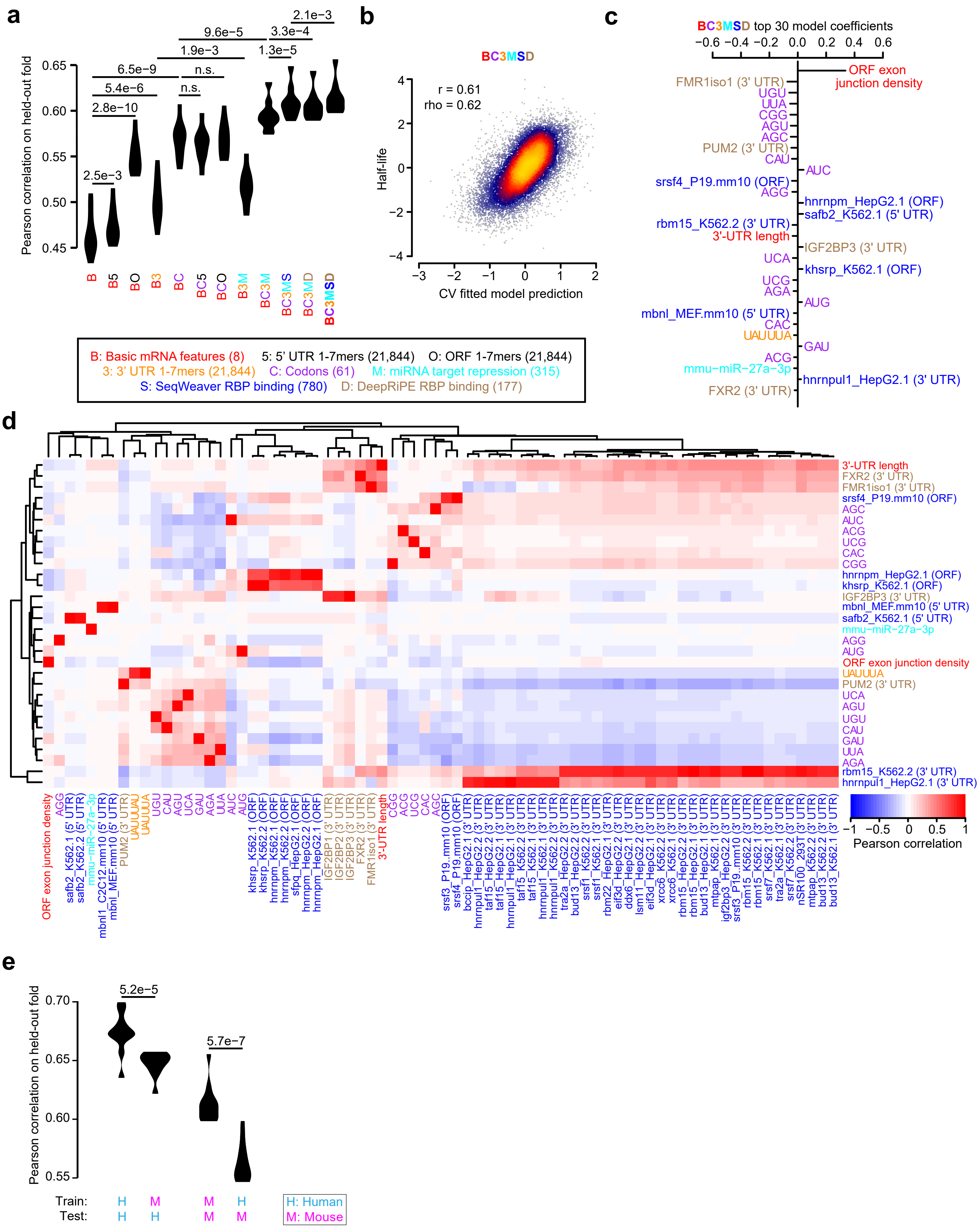
a
Pearson correlation on held-out fold
0.65
0.60
0.55
0.50
0.45
2.5e−3
2.8e−10
5.4e−6
6.5e−9
n.s.
n.s.
1.9e−3
9.6e−5
1.3e−5
3.3e−4
2.1e−3
B
B5
BO
B3
BC
BC5
BCO
B3M
BC3M
BC3MS
BC3MD
BC3MSD
b
BC3MSD
r = 0.61
rho = 0.62
Half-life
CV fitted model prediction
c
BC3MSD top 30 model coefficients
−0.6 −0.4 −0.2 0.0 0.2 0.4 0.6
ORF exon junction density
FMR1iso1 (3' UTR)
UGU
UUA
CGG
AGU
AGC
PUM2 (3' UTR)
CAU
AUC
srsf4_P19.mm10 (ORF)
AGG
hnrnpm_HepG2.1 (ORF)
safb2_K562.1 (5' UTR)
rbm15_K562.2 (3' UTR)
3'-UTR length
IGF2BP3 (3' UTR)
UCA
khsrp_K562.1 (ORF)
UCG
AGA
AUG
mbnl_MEF.mm10 (5' UTR)
CAC
UAUUUA
GAU
ACG
mmu−miR−27a−3p
hnrnpul1_HepG2.1 (3' UTR)
FXR2 (3' UTR)
B: Basic mRNA features (8)
5: 5' UTR 1-7mers (21,844)
O: ORF 1-7mers (21,844)
3: 3' UTR 1-7mers (21,844)
C: Codons (61)
M: miRNA target repression (315)
S: SeqWeaver RBP binding (780)
D: DeepRiPE RBP binding (177)
d
3'-UTR length
FXR2 (3' UTR)
FMR1iso1 (3' UTR)
srsf4_P19.mm10 (ORF)
AGC
AUC
ACG
UCG
CAC
CGG
hnrnpm_HepG2.1 (ORF)
khsrp_K562.1 (ORF)
IGF2BP3 (3' UTR)
mbnl_MEF.mm10 (5' UTR)
safb2_K562.1 (5' UTR)
mmu−miR−27a−3p
AGG
AUG
ORF exon junction density
UAUUUA
PUM2 (3' UTR)
UCA
AGU
UGU
CAU
GAU
UUA
AGA
rbm15_K562.2 (3' UTR)
hnrnpul1_HepG2.1 (3' UTR)
Pearson correlation
−1 −0.5 0 0.5 1
e
Pearson correlation on held-out fold
0.70
0.65
0.60
0.55
5.2e−5
5.7e−7
Train: H M M H
Test: H H M M
H: Human
M: Mouse

Fig S4. Prediction of mouse half-lives using sequence-encoded features.

a-d) These panels are organized in the same fashion as **Fig. 3a-d**, except that they evaluate features benchmarked upon mouse data rather than human data. **e)** Performance of trained human and mouse models on test sets derived from either the same or different species. Due to the imperfect one-to-one miRNA mappings between the human and mouse, miRNA-related coefficients were excluded from the inter-species predictions. Each statistical comparison shown was evaluated with a paired t-test.

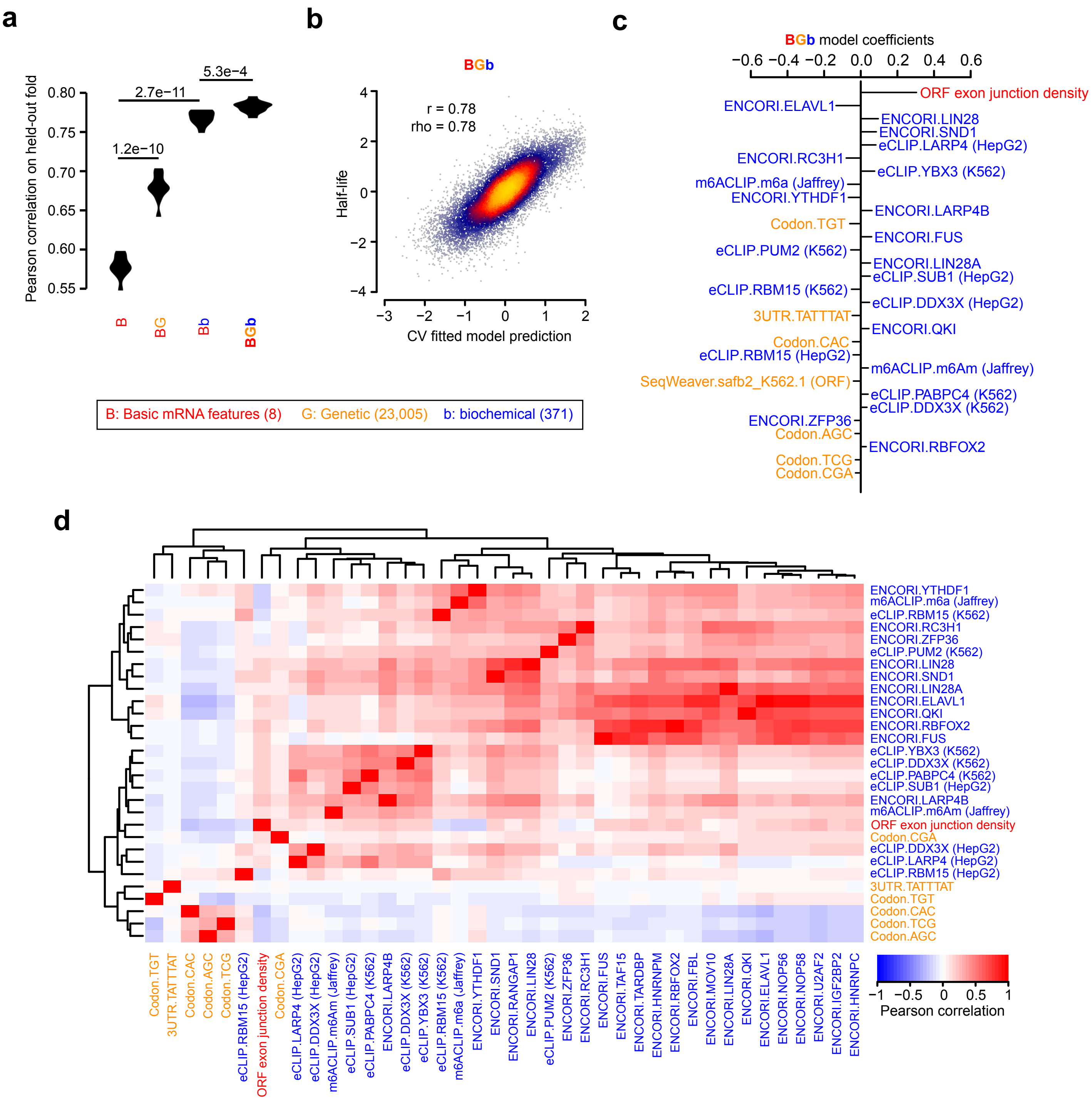

Fig S5. Prediction of mouse half-lives using biochemical and sequence-encoded features.

This figure is organized in the same fashion as **Fig. 3-4**, except that it evaluates the subset of optimal genetic features (BC3MS model, **Fig. 3**), optimal biochemical features (BEeM model, **Fig. 4**), or a combination of both genetic and biochemical features. The features shown in panels (c-d) are described alongside their corresponding source, such as eCLIP, SeqWeaver, or codon features.

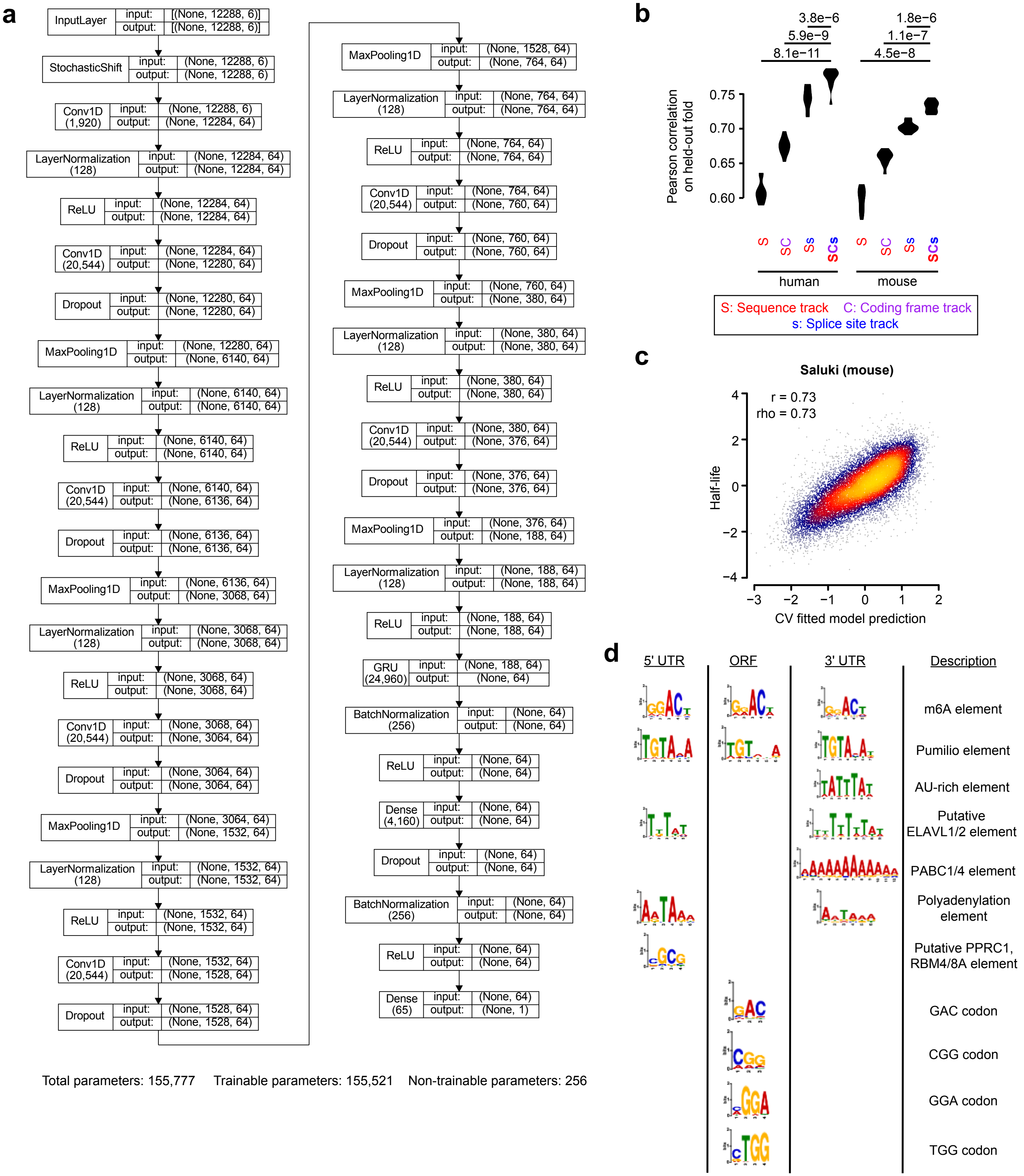
a
InputLayer | input: [(None, 12288, 6)] | output: [(None, 12288, 6)]
StochasticShift | input: (None, 12288, 6) | output: (None, 12288, 6)
Conv1D (1,920) | input: (None, 12288, 6) | output: (None, 12284, 64)
LayerNormalization (128) | input: (None, 12284, 64) | output: (None, 12284, 64)
ReLU | input: (None, 12284, 64) | output: (None, 12284, 64)
Conv1D (20,544) | input: (None, 12284, 64) | output: (None, 12280, 64)
Dropout | input: (None, 12280, 64) | output: (None, 12280, 64)
MaxPooling1D | input: (None, 12280, 64) | output: (None, 6140, 64)
LayerNormalization (128) | input: (None, 6140, 64) | output: (None, 6140, 64)
ReLU | input: (None, 6140, 64) | output: (None, 6140, 64)
Conv1D (20,544) | input: (None, 6140, 64) | output: (None, 6136, 64)
Dropout | input: (None, 6136, 64) | output: (None, 6136, 64)
MaxPooling1D | input: (None, 6136, 64) | output: (None, 3068, 64)
LayerNormalization (128) | input: (None, 3068, 64) | output: (None, 3068, 64)
ReLU | input: (None, 3068, 64) | output: (None, 3068, 64)
Conv1D (20,544) | input: (None, 3068, 64) | output: (None, 3064, 64)
Dropout | input: (None, 3064, 64) | output: (None, 3064, 64)
MaxPooling1D | input: (None, 3064, 64) | output: (None, 1532, 64)
LayerNormalization (128) | input: (None, 1532, 64) | output: (None, 1532, 64)
ReLU | input: (None, 1532, 64) | output: (None, 1532, 64)
Conv1D (20,544) | input: (None, 1532, 64) | output: (None, 1528, 64)
Dropout | input: (None, 1528, 64) | output: (None, 1528, 64)
MaxPooling1D | input: (None, 1528, 64) | output: (None, 764, 64)
LayerNormalization (128) | input: (None, 764, 64) | output: (None, 764, 64)
ReLU | input: (None, 764, 64) | output: (None, 764, 64)
Conv1D (20,544) | input: (None, 764, 64) | output: (None, 760, 64)
Dropout | input: (None, 760, 64) | output: (None, 760, 64)
MaxPooling1D | input: (None, 760, 64) | output: (None, 380, 64)
LayerNormalization (128) | input: (None, 380, 64) | output: (None, 380, 64)
ReLU | input: (None, 380, 64) | output: (None, 380, 64)
Conv1D (20,544) | input: (None, 380, 64) | output: (None, 376, 64)
Dropout | input: (None, 376, 64) | output: (None, 376, 64)
MaxPooling1D | input: (None, 376, 64) | output: (None, 188, 64)
LayerNormalization (128) | input: (None, 188, 64) | output: (None, 188, 64)
ReLU | input: (None, 188, 64) | output: (None, 188, 64)
GRU (24,960) | input: (None, 188, 64) | output: (None, 64)
BatchNormalization (256) | input: (None, 64) | output: (None, 64)
ReLU | input: (None, 64) | output: (None, 64)
Dense (4,160) | input: (None, 64) | output: (None, 64)
Dropout | input: (None, 64) | output: (None, 64)
BatchNormalization (256) | input: (None, 64) | output: (None, 64)
ReLU | input: (None, 64) | output: (None, 64)
Dense (65) | input: (None, 64) | output: (None, 1)
Total parameters: 155,777 Trainable parameters: 155,521 Non-trainable parameters: 256
b
3.8e−6
5.9e−9
8.1e−11
1.8e−6
1.1e−7
4.5e−8
Pearson correlation on held-out fold
0.75
0.70
0.65
0.60
S SC Ss SCs S SC Ss SCs
human
mouse
S: Sequence track C: Coding frame track
s: Splice site track
c
Saluki (mouse)
r = 0.73
rho = 0.73
Half-life
CV fitted model prediction
d
5' UTR
ORF
3' UTR
Description
m6A element
Pumilio element
AU-rich element
Putative ELAVL1/2 element
PABC1/4 element
Polyadenylation element
Putative PPRC1, RBM4/8A element
GAC codon
CGG codon
GGA codon
TGG codon

Fig S6. Performance and interpretation of Saluki.

a) Complete architecture of the Saluki model. Indicated for each layer is the layer name, number of parameters in parentheses when applicable, and dimensionality of the input and output matrices. **b)** Performance of trained Saluki models on each of 10 held-out folds of data. Compared is the relative performance between pairs of models, for both human and mouse species, which iteratively consider additional input tracks. Each model is described by a code indicating the input track considered. A description of the code is provided in the key. An improvement in a more complex model relative to a simpler model was evaluated with a one-sided, paired t-test, adjusted with a Bonferroni correction to account for the total number of hypothesis tests. **c)** This panel is organized in the same fashion as **Fig. 5c**, except that it evaluates the performance on mouse data rather than human data. **d)** Set of enriched motifs discovered by TF-MoDISco [84] in each of the three functional regions of an mRNA.

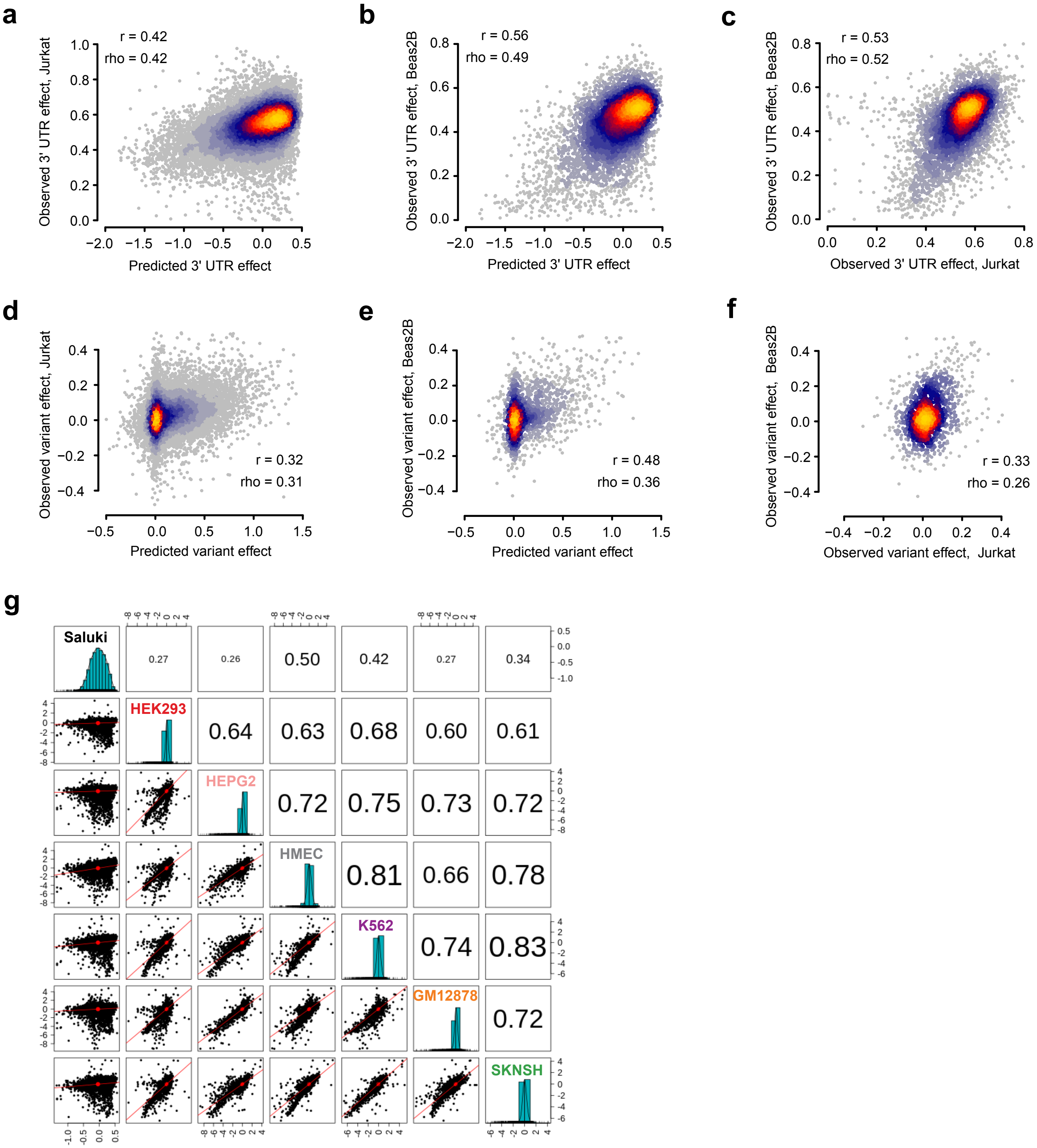
a
r = 0.42
rho = 0.42
Observed 3' UTR effect, Jurkat
Predicted 3' UTR effect
b
r = 0.56
rho = 0.49
Observed 3' UTR effect, Beas2B
Predicted 3' UTR effect
c
r = 0.53
rho = 0.52
Observed 3' UTR effect, Beas2B
Observed 3' UTR effect, Jurkat
d
Observed variant effect, Jurkat
r = 0.32
rho = 0.31
Predicted variant effect
e
Observed variant effect, Beas2B
r = 0.48
rho = 0.36
Predicted variant effect
f
Observed variant effect, Beas2B
r = 0.33
rho = 0.26
Observed variant effect, Jurkat
g
Saluki
0.27
0.26
0.50
0.42
0.27
0.34
HEK293
0.64
0.63
0.68
0.60
0.61
HEPG2
0.72
0.75
0.73
0.72
HMEC
0.81
0.66
0.78
K562
0.74
0.83
GM12878
0.72
SKNSH

Fig S7. Concordance of Saluki predictions and additional functional data from massively parallel reporter assays.

a-c) Scatter plot of the observed and predicted 3′-UTR effects, as measured in a) Jurkat cells or b) Beas2B cells, alongside c) a plot of observed 3′-UTR effects between the pair of cell types [88]. **d-f)** These panels are organized like panels (a-c), but display variant effects instead of 3′-UTR effects [88]. Panel e) is identical to that shown in **Fig. 6e** and reproduced here for convenience. Also indicated are the Pearson (r) and Spearman (rho) correlation values for panels (a-f). **g)** Scatter matrix displaying scatter plots corresponding to each of the 21 pairs of possible comparisons (lower diagonal elements) involving Saluki predictions as well as the measured stability effects for 3′-UTR fragments measured in each of six cell types [89]. Shown on the diagonal is a histogram of the predicted or observed RNA half-life scores. Also shown are Spearman correlation values among each pair of comparisons, with the size of the text proportional to the magnitude of the correlation coefficient (upper diagonal elements).

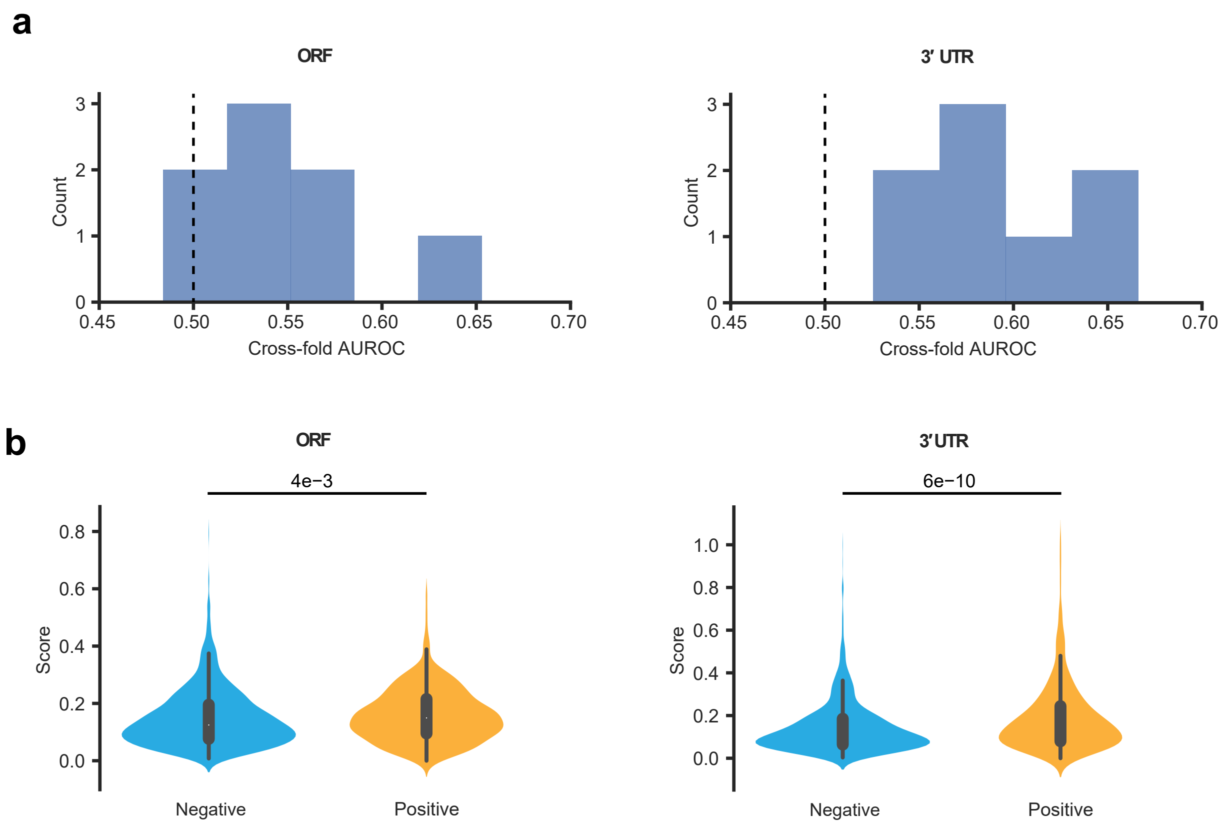

Fig S8. Performance of Saluki on the prediction of fine-mapped eQTLs.
a) Performance of Saluki predictions in classifying likely causal eQTLs from a negative set of gene-expression-level-matched eQTLs that are unlikely to be causal. Plotted are histograms showing the AUROCs for each of 8 folds of data for both ORF (n=352) and 3′-UTR (n=541) variants. Both distributions are significantly greater than the expected performance of a random predictor (dashed line); with p-values of 3.5e-2 and 1e-4 for ORFs and 3′ UTRs, respectively, as evaluated using a binomial test. **b)** Violin plots showing the distributions of the absolute values of the change in predicted Saluki scores for the mutant and wild-type allele, corresponding to both the positive and negative sets of likely causal variants. Also shown are p-values indicating the statistical differences between distributions as evaluated using a two-sided Wilcoxon rank-sum test.
