# Supplementary material for: The genetic and biochemical determinants of mRNA degradation rates in mammals
Source: Genome Biol. 2022 Nov 23;23:245. doi: 10.1186/s13059-022-02811-x (PMC9684954; doi:10.1186/s13059-022-02811-x)
Supplement: Supplementary file 4 — Additional file 4. Review history. [file 13059_2022_2811_MOESM4_ESM.docx]

Review History

**First round of review**

**Reviewer 1**

**Were you able to assess all statistics in the manuscript, including the appropriateness of statistical tests used?** Yes, and I have assessed the statistics in my report.

**Comments to author:**

In this work, the authors first construct a compendium of all published reports detailing the genome-wide set of mRNA half-life measurements collected in vivo from human and mouse samples. These mRNA half-life measurements were obtained using either transcription shut-off or pulse-chase experimental set-ups. A meta-analysis was conducted on these datasets, which showed that the results from these experiments clustered mainly by experimental set-up. This was to be expected, as detailed in the Introduction. After rigorously normalizing the data from each experiment, the authors constructed a suite of ML/DL models. The first models were built using genetically encoded elements and the second set of models were built using in vivo biochemical data extracted from several known RBP binding site repositories. These two sets of models were used to determine which sequence features / RBPs have the greatest impact on global mRNA half-lives. While these models did not yield many new mRNA stability determinants, they were able to rank the impact of each determinant on mRNA stability, which to my knowledge has not been done before. Their third model, which they named Saluki, utilized a more sophisticated computational framework than the previous two sets of models. Saluki was built using mRNA sequences combined with the relative positions of splice sites and the start/stop codons. Saluki was shown to be a 50% improvement to other previously reported models built from human data. In addition, Saluki was shown to benchmark well to previously conducted MPRAs. Using ISM, Saluki was used to predict the positional importance of many genetically encoded stability determinants, which was also very interesting. Saluki will allow researchers to predict the impact of any genetic mutation, inserted into any position, on the stability of a target mRNA. The results of this work, and the tools presented therein, will be of interest to the computational, engineering, and mRNA biology communities. In conclusion, I suggest that this work be published in Genome Biology after several revisions, detailed below.

Major Points:

* Considering that 15 samples were excluded from the final analyses, it should be specified in the abstract that your models were built using 39 samples, rather than 54.

* Why were computationally predicted RBP binding sites used to build the genetic model when the biochemical model was built using a comprehensive suite of in vivo RBP binding site data. In addition, why were basic mRNA features included in the biochemical model if these features are genetically encoded? Would the inclusion of identical features, especially features as fundamental as 'basic mRNA features' impact the ability of each model to accurately rank the relative importance of other non-redundant features? The rationale behind this redundancy of features should be elaborated.

* In either the Results or the Conclusions section of the Abstract, please include more details pertaining to the new biology identified by these models.

* The primary metric that the authors use to quantify model performance is r-value. There were many cases where one model was said to outperform another model based on a seemingly marginal increase in r-value. Are these increases significant on a biological level? I see that an important output of each model is a ranking of determinant coefficients. How different are the rankings provided by models with r-values that differ by 0.1 vs. the rankings of models that differ by 0.01? If we define the difference in r-value between two models as Δr-value, do the coefficient rankings get increasingly divergent as a function of Δr-value?

* The authors found that the human genetic model applied to the mouse half-life data yielded an r-value of ~0.55 (based on Figure S4). This would be an r2 of ~0.30. The authors conclude from these analyses that these models can be used to predict half-lives across the mammalian phylogeny. Considering that the authors begin the Introduction by stating that r2 values of lower than 0.39 are not highly predictive, the language used to describe this result should be softened.

* Can Saluki be applied to non-human datasets? How does it perform using mouse half-life data?

Minor Points:

* 'Integrating the modeling of transcription and RNA decay into a unified model to predict steady-state mRNA levels [2] from genetic sequences would elucidate the full spectrum of regulatory functions of non-coding DNA.' This sentence needs a little more elaboration. Are you referring to ncDNA that is transcribed? not transcribed? Both?

* In the Introduction, please be cognizant of the usage of 'RNA' vs. 'mRNA'. In some cases, the authors use 'RNA' where 'mRNA' would be more appropriate.

* Please define 'ORF exon junction density' at its first mention.

* According to the authors, their combined biochemical and genetic model was the most predictive of mRNA half-lives (highest r-value) out of all their models. I believe that this warrants a few additional sentences pertaining to the findings of this model.

**Reviewer 2**

**Were you able to assess all statistics in the manuscript, including the appropriateness of statistical tests used?** Yes, and I have assessed the statistics in my report.

**Comments to author:**

Summary: The authors perform a meta-analysis of 54 human and 27 mouse datasets of mRNA half-life measurements, including datasets generated from transcriptional inhibition experiments (e.g. based on ActD) and from pulse-chase based metabolic labeling methods (e.g. 4sU and BrU). The authors quantify substantial measurement bias (and batch effects) and hence apply rigorous data processing techniques to both impute missing gene measurements from individual datasets and to estimate a single consensus half-life measurement for each gene across all experiments. The authors proceed by training sparse regression models (LASSO) on both sequence-derived features (e.g. GC content, length, UTR k-mers and predicted RBP binding scores) and biochemical features (e.g. CLIP and eCLIP measurements of RBP binding events) in order to predict the consensus half-life measurements. The authors learn predictive features which align well with the current understanding of cis- and trans-regulatory effects on mRNA stability (e.g. ARE- and PUF binding motifs; please see below for questions about the discovered polyadenylation element as a predictive feature of half-life). By training a neural network on one-hot encoded representations of the mRNA transcripts to predict half-lifes, the authors arrive at a model more accurate than any previous model of mRNA stability (r = 0.77). In-silico interpretation methods (ISM and motif insertions) recapitulate the most important motifs found by the linear regression model, with additional quantification of position-specific effects. Finally, the authors demonstrate good performance at the task of predicting 3' UTR variant effects as measured in MPRA experiments (e.g. r = 0.60 on 3,000 UTRs profiled by fastUTR).

Use of statistics: Appropriate correlation metrics (e.g. pearson r or spearman r) are used throughout the paper and are reported with either p-values or sample sizes. When evaluating model performances for feature selection, the authors make use of appropriate statistical tests (one-sided t-test) for comparisons.

Figure quality: The figures are of good quality. The illustrations are easy to grasp. Statistics such as correlation metrics and exact sample sizes are reported in figures where needed. Figure captions are legible. The captions could benefit from being a bit more concise, with some details moved to methods or the main text.

Significance: The paper represents a significant advancement in the ability to infer mRNA half-life in mammals. There are two main contributions of this paper: (1) The authors perform a very comprehensive meta-analysis of more or less all existing mRNA decay datasets, hence obtaining consensus half-life estimates that are likely the most accurate to date, and (2) the authors learn a sequence-predictive model with proven variant effect scoring performance (on MPRA data), making it an important tool for assessing the impact of mutations. One concern is the lack of validation on variation data occurring in endogenous contexts (e.g. eQTLs) and I recommend adding this analysis to strengthen the model credibility. I recommend accepting the paper after revision based on the suggestions below.

Comments and suggested improvements to the paper:

1. While it is comforting that the consensus half-life measurements (PC1 from the corrected gene x sample matrix) remain strongly correlated between human and mouse, it does not necessarily mean that the ultimate model performance would not improve by also including PC2 (or PC3, etc.). I suggest performing additional analysis (at least for the supplement) showing that Saluki's CV performance (or even better, downstream MPRA variant effect prediction performance) decreases by including PC2 in the consensus measurements.

2. It is hard for the reader to assess (or guess) how well Saluki's performance generalizes from MPRA-based variant effects to variation measured in the genome (e.g. eQTLs). I suggest performing additional analysis comparing Saluki variant effect predictions to a selection of eQTL effect size estimates (e.g. on Lead SNPs in the GTEx cohort).

3. It would be appropriate to include a benchmark comparison against any other predictive model of mRNA half-life on the 3' UTR MPRA variants in Figure 6, or at the very least a baseline comparison against CADD scores or conservation scores (e.g. PhyloP, PhastCons).

4. It is unclear to me why the regression model (and Saluki) is learning the core polyadenylation element AATAAA as a predictive feature of half-life. The authors do not elaborate on this. Is it because of direct half-life effects (e.g. subcellular localization) or because of indirect half-life effects (e.g. the model learns to identify the most abundant APA isoform, which indirectly affects mRNA stability through exclusion of stability elements)? It would strengthen the credibility in the model by answering this question, or at least elaborate on the possible pathways AATAAA may be acting through. For example, using either APA MPRA data or tandem 3' UTR APA events from an endogenous database such as PolyADB (or even just in-silico experiments of synthetically constructed sequences), the authors might investigate whether their predictions correlate with polyadenylation preference.

**Reviewer 3**

**Were you able to assess all statistics in the manuscript, including the appropriateness of statistical tests used?** No, I do not feel adequately qualified to assess the statistics.

**Comments to author:**

In this manuscript, the authors build a compendium of transcriptome-wide mRNA degradation rates in human and mouse cells. They develop an approach to unify measurements of mRNA degradation from different experimental methodologies, cell-types and labs, and produce consensus measurements of mRNA degradation. Using the consensus measurements, they study different properties that affect mRNA degradation, including genetic and biochemical properties, and develop a deep learning neural network model that predicts mRNA degradation based on mRNA sequences, and use it to predict the impact of genetic variations on mRNA stability.

This work provides a resource for mRNA degradation, and a model that can be used to generate predictions of mRNA stability and of the effect of different genetic variations on this property. This model can also provide system-level insights on global genetic and biochemical properties that affect mRNA degradation for further investigation.

However, there are also several concerns that need to be addressed:

1. Introduction (p. 3). The authors describe two strategies to measure mRNA degradation: transcriptional inhibition and metabolic labeling pulse-chase. However, several of the datasets used in this study (Dolken, Bartel, Cramer, Regev datasets and possibly others) rely on metabolic labeling pulse without any chase. The authors should include description of this approach as well, since its used in their compendium and is different than pulse-chase based approaches.

2. Imputing missing half-life predictions (p. 9). If I understand correctly, this procedure relies on half-life prediction from other studies to impute missing predictions in some studies. I expect this procedure would both artificially increase the correlation between studies and obscure differences between cell-types (since imputing is based on other studies done on different cell types). The subsequent observations that cell-type specific effects are not captured (p. 11) and a higher than previously reported correlation between species (p. 12) could be biased by this analysis. Can the authors perform a similar analysis only on genes that were actually measured in all datasets?

3. Unified degradation rates (p. 12). The authors describe the application of a PCA decomposition to the matrix of genes x samples, in which (if I understand correctly) a sample represent a half-life prediction from one dataset. They use the first PC of this analysis as a "unified" degradation rate. However, the authors should provide evidence and justification for selecting the first PC in a PCA analysis of this matrix as a unified measure of mRNA half-lives in their compendium. Including: why this is a valid predictor of half-life? How robust this prediction is? how is it changing if they omit a subset of samples from their analysis?

4. Modeling and predicting degradation rates (p. 13). Several questions arise:

- What are the consequences of using a "unified" degradation estimation for training the model? What kind of cell-type specific information could be overlooked by this approach?

- Do predictions by the model improved when using it to predict specific measurements from a single study rather than the "unified" half-lives?

- Does the machine learning approach identify different or additional features when trained on a specific measurement from a single study? Could it recover cell-type specific effects, for example? Could it be used for cross validation (training on half-lives from one study, and testing on other studies)?

5. Features considered in models (p. 14). Did the authors attempt to include any features that relate to the secondary structure of the mRNA (predicted or experimentally measured)? This could also affect mRNA stability, obscure binding sites etc.

6. Comparison to MPRA (p. 27). The authors claim a "general qualitative agreement". However, in figure 6a almost half of the peaks seem to disagree. A more quantitative comparison between peaks could provide a stronger support for the model.

7. If I understand correctly, the authors refer to their matrix of genes x half-life predictions as "genes x samples" (p. 9, p. 12). This terminology is a bit confusing, and I suggest changing it to something a bit more elaborate

**Authors Response**

**Point-by-point responses to the reviewers’ comments:**

We sincerely appreciate the time and feedback of the reviewers, and are pleased that the reviewers appreciated the scientific advances represented in our work. We sought to further improve the quality of our work using these suggestions, as described in the following point-by-point response:

Reviewer #1: In this work, the authors first construct a compendium of all published reports detailing the genome-wide set of mRNA half-life measurements collected in vivo from human and mouse samples. These mRNA half-life measurements were obtained using either transcription shut-off or pulse-chase experimental set-ups. A meta-analysis was conducted on these datasets, which showed that the results from these experiments clustered mainly by experimental set-up. This was to be expected, as detailed in the Introduction. After rigorously normalizing the data from each experiment, the authors constructed a suite of ML/DL models. The first models were built using genetically encoded elements and the second set of models were built using in vivo biochemical data extracted from several known RBP binding site repositories. These two sets of models were used to determine which sequence features / RBPs have the greatest impact on global mRNA half-lives. While these models did not yield many new mRNA stability determinants, they were able to rank the impact of each determinant on mRNA stability, which to my knowledge has not been done before. Their third model, which they named Saluki, utilized a more sophisticated computational framework than the previous two sets of models. Saluki was built using mRNA sequences combined with the relative positions of splice sites and the start/stop codons. Saluki was shown to be a 50% improvement to other previously reported models built from human data. In addition, Saluki was shown to benchmark well to previously conducted MPRAs. Using ISM, Saluki was used to predict the positional importance of many genetically encoded stability determinants, which was also very interesting. Saluki will allow researchers to predict the impact of any genetic mutation, inserted into any position, on the stability of a target mRNA. The results of this work, and the tools presented therein, will be of interest to the computational, engineering, and mRNA biology communities. In conclusion, I suggest that this work be published in Genome Biology after several revisions, detailed below.

Major Points:

* Considering that 15 samples were excluded from the final analyses, it should be specified in the abstract that your models were built using 39 samples, rather than 54.

Thank you for this note; we have now altered the abstract to reflect a total of 39 samples.

* Why were computationally predicted RBP binding sites used to build the genetic model when the biochemical model was built using a comprehensive suite of in vivo RBP binding site data. In addition, why were basic mRNA features included in the biochemical model if these features are genetically encoded? Would the inclusion of identical features, especially features as fundamental as 'basic mRNA features' impact the ability of each model to accurately rank the relative importance of other non-redundant features? The rationale behind this redundancy of features should be elaborated.

Computationally predicted RBP binding sites were used to build the genetic model because they are a function of only the genetic sequence. In contrast, the biochemical model does not have this requirement, so we included a comprehensive suite of *in vivo* RBP binding site data. The research objective of the biochemical model is to identify biochemical determinants of RNA half-life. The research objective of the genetic model is to benchmark how well RNA half-life can be predicted from the RNA sequence and explore mutation effect prediction.

Basic mRNA features were included in the biochemical model, even though these features are genetically encoded, because they explain a substantial proportion of RNA half-life variance, and the biochemical model would have been more difficult to interpret without them. To provide several examples of why this might be: i) some of the biochemical features such as “number of CLIP peaks for RBP *X*” might trivially correlate with features such as mRNA length or GC content in different functional regions, and ii) an RBP that often binds at splice junctions might have been included in the model instead of simply counting the number of splice junctions. Thus, including these basic features helps to contextualize the true contribution of the biochemical features, and forces the lasso regression model to select them only in the scenario that they contain useful information beyond basic correlates of half-life.

We have now incorporated these lines of reasoning into the main text to further justify our decision-making.

* In either the Results or the Conclusions section of the Abstract, please include more details pertaining to the new biology identified by these models.

We have added the following sentence to the Abstract to highlight what we believe to be the most important novel biological insight of Saluki: “The key novel principle learned by Saluki is that the specific spatial positioning of splice sites, codons, and RNA-binding motifs within an mRNA is strongly associated with mRNA half-life.”

* The primary metric that the authors use to quantify model performance is r-value. There were many cases where one model was said to outperform another model based on a seemingly marginal increase in r-value. Are these increases significant on a biological level? I see that an important output of each model is a ranking of determinant coefficients. How different are the rankings provided by models with r-values that differ by 0.1 vs. the rankings of models that differ by 0.01? If we define the difference in r-value between two models as Δr-value, do the coefficient rankings get increasingly divergent as a function of Δr-value?

We are not sure exactly how to assess “biological significance”, as it seems subjective (i.e., a small statistically significant change could from one perspective carry a significant biological meaning, but from another one not). In figures such as **Fig 3a** and **Fig 4a,** we quantitatively show the precise amounts by which different feature groups enhance model performance, supported by tests for statistical significance.

Although it would be interesting, we do not see a clear path to quantifying how the coefficient rankings vary with model accuracy divergence. We display and examine these rankings in order to highlight features worthy of additional consideration, but we do not recommend interpreting the precise relative rankings, particularly of biochemical features that often correlate with one another.

* The authors found that the human genetic model applied to the mouse half-life data yielded an r-value of ~0.55 (based on Figure S4). This would be an r2 of ~0.30. The authors conclude from these analyses that these models can be used to predict half-lives across the mammalian phylogeny. Considering that the authors begin the Introduction by stating that r2 values of lower than 0.39 are not highly predictive, the language used to describe this result should be softened.

We believe the referee is alluding to this sentence: “Finally, we evaluated the ability of the models trained in each species to generalize to the opposite species. Our interspecies comparisons revealed that models tested in the opposite species performed competitively, albeit slightly worse, than models trained within the same species, for both the human and mouse (**Additional file 1: Fig. S4e**). This indicates that the vast majority of the learned half-life-associated features have strong predictive value more generally across the mammalian phylogeny.”

The specific claim we are alluding to relates to the fact that a model trained and tested on the same species is only modestly better than one trained in the opposite species but tested on the identical test set. Nevertheless, we acknowledge that the performance of the mouse model was weaker than that of the human model in absolute terms, and have weakened the language of the final sentence to: “This indicates that the learned half-life-associated features have predictive value more generally across the mammalian phylogeny.”

* Can Saluki be applied to non-human datasets? How does it perform using mouse half-life data?

Saluki was already independently trained and tested in mouse data. Specifically, **FigS4b** demonstrated that the mouse model achieves a Pearson r = 0.61, and **S4e** demonstrates how the mouse model generalizes to the human.

Minor Points:

* 'Integrating the modeling of transcription and RNA decay into a unified model to predict steady-state mRNA levels [2] from genetic sequences would elucidate the full spectrum of regulatory functions of non-coding DNA.' This sentence needs a little more elaboration. Are you referring to ncDNA that is transcribed? not transcribed? Both?

The reference provided (Agarwal & Shendure 2020) attempted to learn features from mammalian promoters, combine them with simple mRNA half-life features, and predict steady-state levels from RNA-seq data. We have added to this sentence as follows to improve the clarity of our intended meaning:

“Integrating the modeling of transcription and RNA decay into a unified model to predict steady-state mRNA levels [2] from genetic sequences would elucidate the full spectrum of regulatory functions of untranslated regions (UTRs) within mRNA, synonymous codon mutations, and non-coding DNA regions such as promoters.”

* In the Introduction, please be cognizant of the usage of 'RNA' vs. 'mRNA'. In some cases, the authors use 'RNA' where 'mRNA' would be more appropriate.

We have now replaced each instance of RNA with mRNA, aside from several cases that apply more universally to all types of RNA.

* Please define 'ORF exon junction density' at its first mention.

Thank you, we have now defined it in the main text in its first instance.

* According to the authors, their combined biochemical and genetic model was the most predictive of mRNA half-lives (highest r-value) out of all their models. I believe that this warrants a few additional sentences pertaining to the findings of this model.

The text was augmented to include this description of the key findings of our joint model: “The biochemical features were dominantly used in this model, although several codons were selected, along with the ARE 7-mer, which minimized the effect size of the coefficient attributable to ELAVL1 (**Additional file 1: Fig. S5c,d**). Putative novel regulators which emerged in the top features of this analysis include QKI, m6Am, and ZFP36, with most of the relative feature rankings similar to those previously detected.”

Reviewer #2: Summary: The authors perform a meta-analysis of 54 human and 27 mouse datasets of mRNA half-life measurements, including datasets generated from transcriptional inhibition experiments (e.g. based on ActD) and from pulse-chase based metabolic labeling methods (e.g. 4sU and BrU). The authors quantify substantial measurement bias (and batch effects) and hence apply rigorous data processing techniques to both impute missing gene measurements from individual datasets and to estimate a single consensus half-life measurement for each gene across all experiments. The authors proceed by training sparse regression models (LASSO) on both sequence-derived features (e.g. GC content, length, UTR k-mers and predicted RBP binding scores) and biochemical features (e.g. CLIP and eCLIP measurements of RBP binding events) in order to predict the consensus half-life measurements. The authors learn predictive features which align well with the current understanding of cis- and trans-regulatory effects on mRNA stability (e.g. ARE- and PUF binding motifs; please see below for questions about the discovered polyadenylation element as a predictive feature of half-life). By training a neural network on one-hot encoded representations of the mRNA transcripts to predict half-lifes, the authors arrive at a model more accurate than any previous model of mRNA stability (r = 0.77). In-silico interpretation methods

(ISM and motif insertions) recapitulate the most important motifs found by the linear regression model, with additional quantification of position-specific effects. Finally, the authors demonstrate good performance at the task of predicting 3' UTR variant effects as measured in MPRA experiments (e.g. r = 0.60 on 3,000 UTRs profiled by fastUTR).

Use of statistics: Appropriate correlation metrics (e.g. pearson r or spearman r) are used throughout the paper and are reported with either p-values or sample sizes. When evaluating model performances for feature selection, the authors make use of appropriate statistical tests (one-sided t-test) for comparisons.

Figure quality: The figures are of good quality. The illustrations are easy to grasp. Statistics such as correlation metrics and exact sample sizes are reported in figures where needed. Figure captions are legible. The captions could benefit from being a bit more concise, with some details moved to methods or the main text.

Significance: The paper represents a significant advancement in the ability to infer mRNA half-life in mammals. There are two main contributions of this paper: (1) The authors perform a very comprehensive meta-analysis of more or less all existing mRNA decay datasets, hence obtaining consensus half-life estimates that are likely the most accurate to date, and (2) the authors learn a sequence-predictive model with proven variant effect scoring performance (on MPRA data), making it an important tool for assessing the impact of mutations. One concern is the lack of validation on variation data occurring in endogenous contexts (e.g. eQTLs) and I recommend adding this analysis to strengthen the model credibility. I recommend accepting the paper after revision based on the suggestions below.

Comments and suggested improvements to the paper:

1. While it is comforting that the consensus half-life measurements (PC1 from the corrected gene x sample matrix) remain strongly correlated between human and mouse, it does not necessarily mean that the ultimate model performance would not improve by also including PC2 (or PC3, etc.). I suggest performing additional analysis (at least for the supplement) showing that Saluki's CV performance (or even better, downstream MPRA variant effect prediction performance) decreases by including PC2 in the consensus measurements.

We trained a new set of multi-task Saluki models using PCs 1, 2, and 3 for each gene. Our test set accuracy for PC1 did not significantly increase, changing from 0.754 to 0.756 in the human (non-significant t-test p-value of 0.13) and 0.713 to 0.715 (non-significant t-test p-value of 0.06) in the mouse. This result indicated that the additional PCs do not help learn any more about the first. Second, the models do learn to explain variance in PC2 and PC3; test set PearsonR is 0.634 for PC2 and 0.372 for PC3. This indicates that these PC values do depend on transcript sequence, but they still may describe biologically irrelevant bias.

As far as we can tell, that is the case; we could not find any evidence that PC2 or 3 contain biologically relevant information. For example, performance on a new eQTL classification

benchmark (shown in **Fig. S8**) is unaffected by including variant effect predictions for PCs 2 and 3 (AUROC 0.573 versus 0.575; t-test across eight folds p-value 0.91). Aside from this analysis, we followed the reviewer’s suggestion of evaluating downstream MPRA data while considering the predictions of other PCs. We reproduced **Fig. 6e**, which evaluates variant effects measured in Beas2B cells. We computed adjusted R^2^ values and achieved the following results after fitting a multiple linear regression model between our predictors and the observed data:

Adjusted R^2^: 0.2239 (PC1 only)

Adjusted R^2^: 0.2259 (PC1 and PC2)

Adjusted R^2^: 0.2277 (PC1, PC2, and PC3)

As we can see, the improvement of the R^2^ was vanishingly small (~0.002) after considering PC2 and PC3 in conjunction with PC1. We have therefore opted to maintain our current framework of focusing on PC1 in the paper, while adding a few lines in the **Methods** section to summarize our findings:

“During the course of this study, we also explored the utility of PC2 and PC3. Although we found that our Saluki model could learn to explain variance in these PCs (Pearson correlation of 0.634 and 0.372 for PC2 and PC3, respectively), we were unable to detect biologically relevant signals in these PCs, and found that they did not appear to improve downstream variant effect prediction tasks.”

2. It is hard for the reader to assess (or guess) how well Saluki's performance generalizes from MPRA-based variant effects to variation measured in the genome (e.g. eQTLs). I suggest performing additional analysis comparing Saluki variant effect predictions to a selection of eQTL effect size estimates (e.g. on Lead SNPs in the GTEx cohort).

We have now performed an analysis using Saluki predictions to classify likely causal eQTLs from expression-matched, likely non-causal eQTLs overlapping ORFs and 3’ UTRs (presented in our new **Fig. S8**, shown below for convenience). We have accordingly augmented the text of our results section and methods to reflect our new analysis.

**Figure S8. Performance of Saluki on the prediction of fine-mapped eQTLs.**

**a)** Performance of Saluki predictions in classifying likely causal eQTLs from a negative set of gene-expression-level-matched eQTLs that are unlikely to be causal. Plotted are histograms showing the AUROCs for each of 8 folds of data for both ORF (n=352) and 3′-UTR (n=541) variants. Both distributions are significantly greater than the expected performance of a random predictor (dashed line); with p-values of 3.5e-2 and 1e-4 for ORFs and 3′ UTRs, respectively, as evaluated using a binomial test. **b)** Violin plots showing the distributions of the absolute values of the change in predicted Saluki scores for the mutant and wild-type allele, corresponding to both the positive and negative sets of likely causal variants. Also shown are p-values indicating the statistical differences between distributions as evaluated using a two-sided Wilcoxon rank-sum test.

3. It would be appropriate to include a benchmark comparison against any other predictive model of mRNA half-life on the 3' UTR MPRA variants in Figure 6, or at the very least a baseline comparison against CADD scores or conservation scores (e.g. PhyloP, PhastCons).

We have now added the following excerpt to compare **Fig. 6c** against the recommended baselines:

“Collectively, the variant effect predictions agreed with those observed with a Spearman correlation of 0.69 (**Fig. 6c**), marginally better than the corresponding correlation of 0.64 achieved by CADD v1.6 [89]. We also compared our performance to that of scores from 100-way phyloP [90], which does not distinguish between alternative SNPs in each position.

While phyloP achieved a Spearman correlation of 0.26 to the mean mutation effect size among positions, Saluki achieved a correlation of 0.80.”

Several properties of the variant data presented in **Fig 6e**, derived from Siegel et al, 2022, make it impossible to directly compare Saluki performance to the recommended baselines. Specifically: i) many of the variants tested in the MPRA represent variable-length indels rather than SNPs, and ii) many of the elements tested were tested with simultaneous mutations in multiple AU-rich elements. Unlike Saluki, which can accommodate arbitrary types of mutations, both CADD and phyloP model single nucleotide effects rather than the combinatorial effects of multiple SNPs/indels evaluated in the Siegel et al MPRA. Therefore, it is impossible to calculate a predicted effect for each of the mutations tested or treat them as directly comparable baselines.

4. It is unclear to me why the regression model (and Saluki) is learning the core polyadenylation element AATAAA as a predictive feature of half-life. The authors do not elaborate on this. Is it because of direct half-life effects (e.g. subcellular localization) or because of indirect half-life effects (e.g. the model learns to identify the most abundant APA isoform, which indirectly affects mRNA stability through exclusion of stability elements)? It would strengthen the credibility in the model by answering this question, or at least elaborate on the possible pathways AATAAA may be acting through. For example, using either APA MPRA data or tandem 3' UTR APA events from an endogenous database such as PolyADB (or even just in-silico experiments of synthetically constructed sequences), the authors might investigate whether their predictions correlate with polyadenylation preference.

Mutation effect predictions from our model that break the core polyadenylation element cannot be fully trusted. In reality, breaking this element would likely extend the 3’ UTR to include more sequence, but the model is not able to consider this outcome. Instead, the model is only able to examine transcripts in the training data that have the element versus those that do not. Thus, we believe the reason that the deep learning model has learned the relationship to AAUAAA frequency is the same that our lasso regression model has learned the same property. Namely that in our training set, the frequency of these AAUAAA k-mers in 3’ UTRs is positively associated with half-life (Spearman rho = 0.10, Pearson r = 0.14, p < 1e-16), and is still positively associated with half-life after considering many other sequence features (**Fig 3c**).

We now speculate a reason for why this could be in our revised text: “A potential explanation for the emergence of AAUAAA as a predictive feature is that its presence is associated with the choice of a proximal 3′-UTR isoform, leading to 3′-UTR shortening and the subsequent evasion of binding to repressive RBPs (Mayr and Bartel 2009).”

Reviewer #3: In this manuscript, the authors build a compendium of transcriptome-wide mRNA degradation rates in human and mouse cells. They develop an approach to unify measurements of mRNA degradation from different experimental methodologies, cell-types and labs, and produce consensus measurements of mRNA degradation. Using the consensus measurements,

they study different properties that affect mRNA degradation, including genetic and biochemical properties, and develop a deep learning neural network model that predicts mRNA degradation based on mRNA sequences, and use it to predict the impact of genetic variations on mRNA stability.

This work provides a resource for mRNA degradation, and a model that can be used to generate predictions of mRNA stability and of the effect of different genetic variations on this property. This model can also provide system-level insights on global genetic and biochemical properties that affect mRNA degradation for further investigation.

However, there are also several concerns that need to be addressed:

1. Introduction (p. 3). The authors describe two strategies to measure mRNA degradation: transcriptional inhibition and metabolic labeling pulse-chase. However, several of the datasets used in this study (Dolken, Bartel, Cramer, Regev datasets and possibly others) rely on metabolic labeling pulse without any chase. The authors should include description of this approach as well, since its used in their compendium and is different than pulse-chase based approaches.

We thank the reviewer for noticing our mistake in distinguishing these types of assays. We have now edited the introduction and discussion to indicate that we intended to distinguish pulse-labeling based methods, of which pulse-chase is one form, from transcriptional shutoff-based methods.

2. Imputing missing half-life predictions (p. 9). If I understand correctly, this procedure relies on half-life prediction from other studies to impute missing predictions in some studies. I expect this procedure would both artificially increase the correlation between studies and obscure differences between cell-types (since imputing is based on other studies done on different cell types). The subsequent observations that cell-type specific effects are not captured (p. 11) and a higher than previously reported correlation between species (p. 12) could be biased by this analysis. Can the authors perform a similar analysis only on genes that were actually measured in all datasets?

It is important to note that the results presented in **Fig 1** and **Fig S2b** reflect the pairwise Spearman correlations between genes *prior* to the imputation procedure being run. Therefore the imputation procedure does not artificially inflate the correlations presented in those figures.

However, it is indeed possible that imputation impacts the analyses presented in **Fig 2c** (i.e., the human analysis described previously in p. 11). Thus, we have repeated the analysis with pairwise correlations observed between the measured values (i.e., not imputed values as before) and acquired the following result:

Here, Pearson correlations are plotted on the y-axis as shown before. The “diff” category alludes to the distribution of pairs of samples derived from a different cell type and different study, and “same” category alludes to the distribution of pairs of samples derived from the same cell type but different study. As the reviewer predicted, the correlations are globally lower for both categories; however, there is still no observable cell-type specific signal in the human data as a whole, preserving the original conclusion. We have therefore added the following sentence to the main text to reflect the robustness of the result when tested under these conditions:

“To account for the possibility that our imputation procedure influenced these results, we repeated these analyses by computing correlations from the subset of genes measured between each pair of samples (i.e., prior to imputation). Although the magnitudes of the correlations were slightly lower for all sample pairs, we again observed no cell-type-specific signal for half-lives in human cells.”

3. Unified degradation rates (p. 12). The authors describe the application of a PCA decomposition to the matrix of genes x samples, in which (if I understand correctly) a sample represent a half-life prediction from one dataset. They use the first PC of this analysis as a "unified" degradation rate. However, the authors should provide evidence and justification for selecting the first PC in a PCA analysis of this matrix as a unified measure of mRNA half-lives in their compendium. Including: why this is a valid predictor of half-life? How robust this prediction is? how is it changing if they omit a subset of samples from their analysis?

The first PC of the full mRNA half-life matrix is more meaningfully viewed as a denoised description of mRNA half-life, then as a “predictor”. The first mRNA PC is very similar to simply taking the average of all of the half-life experiments, with PC1 achieving a correlation of 0.999 with simply the average half-life. We chose the first PC over this simpler statistic because many of the datasets belong to the same study and are highly correlated. The first PC implicitly computes a weighted average with weights that are proportional to the inverse of the covariance each sample has with other samples.

Thus, we do not believe asking “how robust this prediction is” is a proper question. However, we can explore the sensitivity to removing samples. We performed an experiment in which we removed 10 random samples (i.e., leaving 29/39 samples) 100 independent times. For each subsample, we recomputed the first PC and computed its correlation to the PC1 derived from all of the samples. This resulted in the following histogram of correlations, which indicates an extremely strong correlation of 0.994 or above in all cases:

Thus, we conclude that PC1 is a robust metric, and have added the following to the main text to further describe it: “When performing robustness tests of PC1 with the human data, we found that it was highly correlated (Pearson correlation > 0.99) to the computation of a gene’s half-life simply as the mean across samples, and was also robust to the loss of random sets of ten samples (Pearson correlation > 0.99, data not shown).”

Finally, we encourage the reviewer to inspect our response to Reviewer 2’s related comments about the use of PC1 relative to other PCs.

4. Modeling and predicting degradation rates (p. 13). Several questions arise: - What are the consequences of using a "unified" degradation estimation for training the model? What kind of cell-type specific information could be overlooked by this approach? - Do predictions by the model improved when using it to predict specific measurements from a single study rather than the "unified" half-lives?

- Does the machine learning approach identify different or additional features when trained on a specific measurement from a single study? Could it recover cell-type specific effects, for example? Could it be used for cross validation (training on half-lives from one study, and testing on other studies)?

Our analysis of the measured RNA half lives across studies revealed no evidence for cell-type specific regulation. Although such regulation clearly exists, these data with the various assay biases do not appear to capture it. Thus, we view the PC1 analysis as a denoising of the measured data to improve the ability for a model to learn the sequence determinants of half life.

During our initial explorations, we trained a multi-task model on the imputed measurements for the 54 human and 27 mouse experiments. Test set Pearson R for these experiments were all less than the test set Pearson R for the PC1 model, indicating that the noise and bias in the individual experiments obstructed effective learning. The figure below is a histogram of the 54 human experiment test Pearson R values and a red line for the PC1 test Pearson R.

Furthermore, the correlations between predictions from these multi-task models roughly reproduce the correlations from the measurements themselves in **Figure 1**, indicating that the originating research lab and assay type are major sources of variance. The clustered heatmap below (next page) shows these correlations:

Finally, we could not find any evidence that these models learn additional cell type specific effects. We also could not find any evidence that individual sample predictions exceed the performance on downstream tasks of our PC1 model. Although one could train on one experiment and test another, we do not see benefits to doing so.

5. Features considered in models (p. 14). Did the authors attempt to include any features that relate to the secondary structure of the mRNA (predicted or experimentally measured)? This could also affect mRNA stability, obscure binding sites etc.

In our initial exploration of this problem, we computed several predicted structural metrics, such as the mean local folding probability of the 5’ UTR, ORF, and 3’ UTR (as evaluated using RNAplfold -L 40 -W 80 -u 20), and did not observe an association to the half-lives beyond the basic mRNA features such as GC content and lengths of different functional regions.

To further demonstrate that RNA structural elements do not measurably improve our model beyond the features already incorporated into the model, we further evaluated a set of 33 RNA structural motifs proposed to regulate mRNA stability (Goodarzi et al, *Nature* 2012, generously provided by H. Goodarzi). We screened these structural motifs across 5’ UTRs, ORFs, and 3’ UTRs to evaluate their significance using pyTEISER (Fish et al, *Science* 2021), and identified a total of 8 structural motifs which were significantly associated to the residuals from our Saluki model. Next, we compared the r^2^ of the observed human half-lives to our Saluki model, either alone or in conjunction with counts for these 8 structural motifs.

Without RNA structural features:

Coefficients:

Estimate Std. Error t value Pr(>|t|)

(Intercept) 0.008319 0.005596 1.487 0.137

Saluki 0.906768 0.006582 137.763 <2e-16 ***

---

Signif. codes: 0 ‘***’ 0.001 ‘**’ 0.01 ‘*’ 0.05 ‘.’ 0.1 ‘ ’ 1

Residual standard error: 0.6372 on 12966 degrees of freedom

Multiple R-squared: 0.5941, Adjusted R-squared: 0.5941

F-statistic: 1.898e+04 on 1 and 12966 DF, p-value: < 2.2e-16

With RNA structural features:

Coefficients:

Estimate Std. Error t value Pr(>|t|)

(Intercept) 0.009204 0.008673 1.061 0.2886

Saluki 0.906993 0.006648 136.433 <2e-16 ***

motif1 -0.006153 0.009498 -0.648 0.5171

motif2 0.008556 0.004785 1.788 0.0738 .

motif3 -0.004027 0.005115 -0.787 0.4312

motif4 0.003360 0.009499 0.354 0.7235

motif5 0.338142 0.201611 1.677 0.0935 .

motif6 -0.005245 0.007162 -0.732 0.4639

motif7 -0.007655 0.008114 -0.943 0.3454

motif8 0.003430 0.007829 0.438 0.6613

---

Signif. codes: 0 ‘***’ 0.001 ‘**’ 0.01 ‘*’ 0.05 ‘.’ 0.1 ‘ ’ 1

Residual standard error: 0.6372 on 12958 degrees of freedom

Multiple R-squared: 0.5944, Adjusted R-squared: 0.5941

F-statistic: 2110 on 9 and 12958 DF, p-value: < 2.2e-16

We observed that the r^2^remained unchanged regardless of the consideration of the structural motifs, with the coefficients associated with the motifs not achieving statistical significance. This indicated that the RNA structural motifs previously claimed to be associated with mRNA stability did not further inform the Saluki model. As a consequence of these observations, we added the following lines to our discussion: “During the course of our work, we also investigated the role of RNA secondary structure in its influence on mRNA stability, as previous work had implicated a role for RNA secondary structural motifs in the regulation of mRNA stability (Goodarzi et al. 2012). However, we found that neither summary metrics of secondary structure (e.g., average predicted structural accessibility) nor the previously implicated structural motifs contributed to improved model performance. These findings suggest that more sophisticated methods of capturing RNA structural features are needed to improve the model, or alternatively that RNA structure might have a modest role with respect to regulating decay due to its largely unfolded state *in vivo* (Rouskin et al. 2014).”

6. Comparison to MPRA (p. 27). The authors claim a "general qualitative agreement". However, in figure 6a almost half of the peaks seem to disagree. A more quantitative comparison between peaks could provide a stronger support for the model.

We had already acknowledged in the text that there are peaks that disagree in the following sentence: “However, there were several novel elements detected by the assay which were conserved but not predicted by the model, indicating several false negatives among the predictions”. We do not claim an overly strong alignment between prediction and observation.

Nevertheless, we agree that this would benefit by a more quantitative comparison, and have therefore modified the aforementioned line to: “We observed a general agreement between model predictions and experiment in the 3′-UTR regions showing high activity, which typically overlapped conserved and ARE-containing regions (**Fig. 6a**, Pearson correlation = 0.29 and Spearman correlation = 0.31 across all positions).”

7. If I understand correctly, the authors refer to their matrix of genes x half-life predictions as "genes x samples" (p. 9, p. 12). This terminology is a bit confusing, and I suggest changing it to something a bit more elaborate

Thank you for the feedback. We have now more explicitly indicated what we mean by this matrix in the following excerpts, and hope it brings clarity to our readers as to what we meant:

“Given the wide disparity in reported genes (**Additional file 1: Fig. S1** and **S2a**) and potential existence of outliers in some samples, we pre-processed our *gene* x *sample* (i.e., representing

*rows* x *columns*) human and mouse half-life matrices to improve our ability to evaluate sample relatedness and examine possible sources of measurement bias.”

And “Finally, we performed PCA on our *sample* x *gene* matrix (i.e., the transpose of our imputed matrix)”

**Second round of review**

**Reviewer 2**

The authors have addressed all my comments

**Reviewer 3**

In this version of the manuscript, the authors have added new analysis and results that make this manuscript clearer and more concise.

In particular, the issues stated in our review questions have been properly addressed, and this additional information has been included in the revised manuscript. In particular, the additional analysis done in response to question #2 improves the confidence in the author’s analysis that no cell-type specific signal for mRNA half-lives in human cells is evident in the data
